# Pitfalls in Suspected Acute Aortic Syndrome: Impact of Appropriate and If Required Repeated Imaging

**DOI:** 10.1155/2015/573256

**Published:** 2015-04-27

**Authors:** C. Meier, M. Lichtenberg, P. Lebiedz, F. Breuckmann

**Affiliations:** ^1^Department of Cardiovascular Medicine, University Hospital Münster, 48149 Münster, Germany; ^2^Department of Angiology, Arnsberg Medical Center, 59759 Arnsberg, Germany; ^3^Department of Cardiology, Arnsberg Medical Center, 59759 Arnsberg, Germany

## Abstract

The incidence of acute aortic syndrome is low, but the spontaneous course is often life-threatening. Adequate ECG-gated imaging is fundamental within the diagnostic workup. We here report a case of a 53-year-old man presenting with atypical chest pain, slight increase of D dimers at admission, and extended diameter of the ascending aorta accompanied by mild aortic regurgitation. Interpretation of an initial contrast-enhanced computed tomography was false negative due to inadequate gating and motion artifacts, thereby judging a tiny contrast signal in the left anterior quadrant of the ascending aorta as a pseudointimal flap. By hazard, cardiac magnetic resonance imaging demonstrated an ulcer-like lesion superior to the aortic root, leading to aortic surgery at the last moment. As sensitivity of imaging is not 100%, this example underlines that second imaging studies might be necessary if the first imaging is negative, but the clinical suspicion still remains high.

## 1. Introduction

The incidence of acute aortic dissection is estimated with 2,9–3,5/100.000 annual lives [1]. The pathogenesis starts with a laceration of the intima, in most cases accompanied by an off-peeling of the inner layer in the direction of the blood flow, so that a false lumen occurs [2]. As to the high mortality and low incidence, especially in type A aortic dissection, diagnosis and management require a fast and multidisciplinary approach, particularly with input from noninvasive imaging techniques in addition to clinical evaluation. Radiologic imaging technologies have improved in terms of detection accuracy in aortic diseases [3–6]. Several factors have an influence on accuracy, for example, scanning-technology, protocol standards, patient's anatomy, and especially the heart rate, producing motion artifacts. As referred to computed tomography (CT), for elimination of this artifact, the ECG-signal is detected synchronously to the acquisition of the CT data. Pictures are generated retrospectively, thereby minimizing artifacts by choosing the most convenient time frame. In contrast, the prospective triggering is an alternative to perform the examination. Using this method the radiation is applied in the expected ideal interval, enabling a reduction of radiation.

## 2. Case Presentation

**Figure 1:**
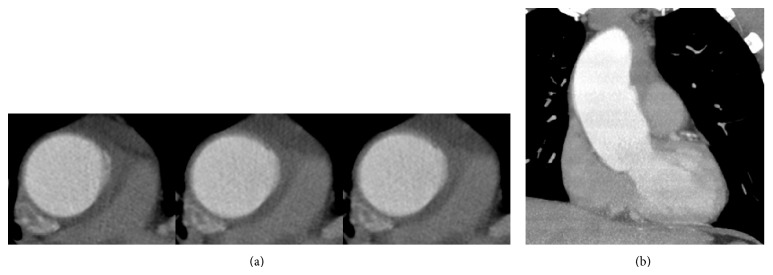
Double-oblique (a) and axial (b) reconstructions (spatial resolution 3 × 0.5 × 0.25 mm) of the initial inadequate ECG-gated contrast-enhanced CT resulting from repeated premature ventricular contractions at the time of image acquisition. Consider the small contrast signal in the left anterior quadrant of the ascending aorta, misdiagnosed as a pseudointimal flap/motion artifact.

A 53-year-old man presented to our emergency department with sudden onset of atypical chest pain and nonsignificant elevated ST-segments in the inferior leads. Baseline myocardial markers were negative and remained at the same level within the follow-up measurements. By contrast, D dimers at admission showed a slight increase (0.7 *μ*g/mL, reference <0.5 *μ*g/mL). Immediately performed transthoracic echocardiography demonstrated a preserved left ventricular global function without significant wall motion abnormalities and no signs of right ventricular strain, though an extended diameter of the ascending aorta of 52 mm was accompanied by a mild aortic regurgitation with hemodynamically noncompromising pericardial effusion. Thus, under the working diagnosis of an acute aortic syndrome, a contrast-enhanced CT using a triple rule out protocol was initiated. Because of inadequate gating due to repeated premature ventricular contractions at the time of image acquisition despite administration of beta-blockers, finally a non-ECG-gated contrast-enhanced CT of the ascending aorta was completed in order to exclude aortic dissection. The CT showed an extended diameter of the ascending aorta with a maximum of 51 mm without hint for dissection membrane or false lumen. However, imaging of the aortic root as well as the supracoronary aorta was hampered by aortic motion artifacts, thereby judging a tiny contrast signal in the left anterior quadrant of the ascending aorta as a pseudointimal flap (Figures 1(a) and 1(b)).

**Figure 2:**
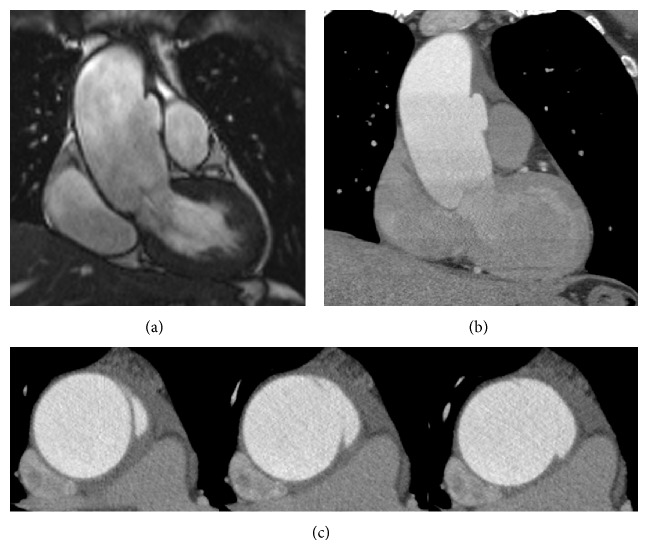
Corresponding double-oblique view of the cine-CMR demonstrating an ulcer-like lesion (acute aortic syndrome, class 4) superior to the aortic root in the even initially suspicious left anterior aortic quadrant (a) and nearly congruent double-oblique (b) and axial (c) reconstructions (spatial resolution 2 × 0.5 × 0.25 mm) as compared to initial CT imaging, this time adequately prospectively ECG-gated. Note the precise demarcation and nearly similar accuracy compared to the preceding CMR of a penetrating aortic ulcer exactly in the same location compared to the initially suspected area.

In order to rule out a peri-/myocarditis as another possible explanation for the persistent clinical symptoms, we performed a cardiac magnet resonance imaging (CMR) shortly after initial presentation. Surprisingly, cine-CMR imaging demonstrated an ulcer-like lesion superior to the aortic root in the left anterior quadrant of the ascending aorta (Figure 2(a)) but no typical late enhancement pattern. A renewed and at this time adequately ECG-gated contrast-enhanced CT revealed a penetrating aortic ulcer exactly in the same location compared to the initially suspected area (Figures 2(b) and 2(c)), thereby showing a nearly similar accuracy as the preceding CMR.

The currently asymptomatic patient was directly admitted to our intensive care. Unfortunately, only a few minutes following the second CT the patient suffered a convulsive seizure with subsequent hemodynamic instability needing cardiopulmonary resuscitation for about 20 minutes until return of spontaneous circulation. The patient was brought immediately to the operation room for aortic surgery. At this time, the operative site demonstrated a supracoronary entry of an extensive classical type A aortic dissection reaching the aortic arch with an inversion of the intima flap that resulted in an occlusion of the supra-aortic limbs. A graft replacement of the ascending aorta under selective cerebral perfusion was done using a 30 mm artificial graft.

Despite the worsening scenario the patient recovered well without relevant neurological residuals until discharge and during subsequent outpatient follow-up consultations.

## 3. Discussion

Type A dissection has a 40–60% risk of death within the first 48 hours. However, not only acute type A dissection but all types of type A acute aortic syndrome require timely diagnosis and early surgery [7]. The classification by Svensson et al. takes account of the precursors of aortic dissection: classic dissection with a correct and a false lumen (class 1), intramural hematoma (class 2), localized dissection with dilatation (class 3), ulcer-like lesion and plaque rupture (class 4), and an iatrogenic or traumatic dissection (class 5) [8]. Urgent imaging of the aorta should be performed by transesophageal echocardiography, CT, or magnetic resonance imaging to identify or exclude thoracic aortic dissection. The selection of a specific imaging should be based on patient's condition as well as institutional capabilities [9]. A second imaging study should be performed if the first imaging is negative, but the clinical suspicion remains probable. As presented in our case, the initial clinical suspicion of acute aortic dissection was right and the interpretation of the presented symptoms was superior to the first CT scan. That points out that anamnesis and exploration should belong to the first important steps on the way to diagnosis. On the other hand, it emphasizes the importance of better correlating imaging data with the clinical picture, which might have led to a different interpretation of the initial CT scan.

Retrospectively, a localized dissection membrane or ulcer-like lesion should have been assumed, but diagnosis failed by insufficient imaging quality and missing of immediate reevaluation. This case should be a reminder that sensitivity of the first performed imaging and particularly CT scan is high but not 100%. Vice versa, the rate of false positive activation remains low and justifiable in such life-threatening disorder [10]. As a result, clinical knowledge about aortic diseases and right interpretation of the symptoms by the physician is fundamental and should in doubt lead to immediate therapy or a second imaging study.
